# Effects of Calorie Restriction and Diet-Induced Obesity on Murine Colon Carcinogenesis, Growth and Inflammatory Factors, and MicroRNA Expression

**DOI:** 10.1371/journal.pone.0094765

**Published:** 2014-04-14

**Authors:** Susan E. Olivo-Marston, Stephen D. Hursting, Susan N. Perkins, Aaron Schetter, Mohammed Khan, Carlo Croce, Curtis C. Harris, Jackie Lavigne

**Affiliations:** 1 Laboratory of Human Carcinogenesis, National Cancer Institute, Center for Cancer Research, National Institutes of Health, Bethesda, Maryland, United States of America; 2 Division of Epidemioogy, The Ohio State University College of Public Health, Columbus, Ohio, United States of America; 3 Department of Nutritional Sciences, University of Texas-Austin, Austin, Texas, United States of America; 4 Department of Molecular Carcinogenesis, University of Texas-MD Anderson Cancer Center, Smithville, Texas, United States of America; 5 Center for Cancer Training, The National Cancer Institute, National Institutes of Health, Bethesda, Maryland, United States of America; 6 Department of Molecular Virology, Immunology and Medical Genetics, The Ohio State University College of Medicine, Columbus, Ohio, United States of America; 7 Division of Cancer Epidemiology and Genetics, National Cancer Institute, NIH, Bethesda, Maryland, United States of America; IRCCS-Policlinico San Donato, Italy

## Abstract

Obesity is an established colon cancer risk factor, while preventing or reversing obesity via a calorie restriction (CR) diet regimen decreases colon cancer risk. Unfortunately, the biological mechanisms underlying these associations are poorly understood, hampering development of mechanism-based approaches for preventing obesity-related colon cancer. We tested the hypotheses that diet-induced obesity (DIO) would increase (and CR would decrease) colon tumorigenesis in the mouse azoxymethane (AOM) model. In addition, we established that changes in inflammatory cytokines, growth factors, and microRNAs are associated with these energy balance-colon cancer links, and thus represent mechanism-based targets for colon cancer prevention. Mice were injected with AOM once a week for 5 weeks and randomized to: 1) control diet; 2) 30% CR diet; or 3) DIO diet. Mice were euthanized at week 5 (n = 12/group), 10 (n = 12/group), and 20 (n = 20/group) after the last AOM injection. Colon tumors were counted, and cytokines, insulin-like growth factor 1 (IGF-1), IGF binding protein 3 (IGFBP-3), adipokines, proliferation, apoptosis, and expression of microRNAs (miRs) were measured. The DIO diet regimen induced an obese phenotype (∼36% body fat), while CR induced a lean phenotype (∼14% body fat); controls were intermediate (∼26% body fat). Relative to controls, DIO increased (and CR decreased) the number of colon tumors (p = 0.01), cytokines (p<0.001), IGF-1 (p = 0.01), and proliferation (p<0.001). DIO decreased (and CR increased) IGFBP-3 and apoptosis (p<0.001). miRs including mir-425, mir-196, mir-155, mir-150, mir-351, mir-16, let-7, mir34, and mir-138 were differentially expressed between the dietary groups. We conclude that the enhancing effects of DIO and suppressive effects of CR on colon carcinogenesis are associated with alterations in several biological pathways, including inflammation, IGF-1, and microRNAs.

## Introduction

Energy balance has been linked with increased risk and/or progression of several different types of cancer, including colon cancer. Chronic positive energy balance, characterized by a higher intake of calories compared to calorie expenditure, results in an overweight or obese phenotype. Many different components of energy balance have been queried in association with cancer including diet- or genetically-induced obesity, total calorie intake, and physical activity, among others [1].

One of the cancer types most consistently associated with obesity is colon cancer. Furthermore, colon cancer incidence tends to be highest in the countries with higher rates of overweight and obesity. Many studies estimate a two-fold increase in colon cancer risk among obese men and a slightly weaker association for women [1], [2], [3], [4]. In addition, body mass index (BMI), the most commonly used measure of obesity, has been associated with increased risk of developing colorectal adenomas [2], [3].

Beginning in the early-to-mid 20^th^ century, when Rous (in 1909) and Tannenbaum (in the 1940's) demonstrated the inhibition of tumors by calorie restriction (CR) [5], animal studies have suggested that dietary energy balance and obesity impacts the carcinogenesis process. Unfortunately, the mechanisms underlying the anticancer effects of CR are poorly characterized. More recent animal studies suggest that CR can inhibit colon tumor development and progression by reducing cellular proliferation and inflammation [6], [7], [8], [9], [10], [11], [12]. CR also inhibits the formation of aberrant crypt foci (ACF), which are preneoplastic lesions associated with increased risk of invasive colon cancer [13]. Epidemiologic data on CR and colon cancer risk is less clear. The majority of the studies have suggested an association between high calorie intake and increased colon cancer risk [14], although not all studies are consistent [15].

The obesity-colon cancer relationship has been less studied in animal models relative to CR. Genetically-induced mouse and rat models of obesity have demonstrated that obesity increases incidence of ACF in the colon, as well as colon adenocarcinomas [16]. Furthermore, diet-induced obesity (DIO) demonstrated an increase in tumor growth rate in a transplant model of murine colon cancer [10], [17].

Taken together, the animal and epidemiologic data support an important link between energy balance and colon cancer. However, a better understanding of the mechanisms underlying the protective effect of CR and the enhancing effects of obesity is urgently needed to identify targets and develop strategies to break the obesity-colon cancer link. One pathway that has emerged in breast, pancreas, prostate, skin, and other tumor model systems is the insulin-like growth factor-1 pathway (IGF-1). CR decreases, while diet- and genetically-induced obesity increases levels of insulin and bioavailable IGF-1 [10], [18]. In addition, human studies have established the positive association between IGF-1 levels and colon cancer risk [19], [20], [21]. Finally, both *in vitro* and animal studies demonstrate the ability of IGF-1 to increase colon tumor cell growth [22], [23], [24].

Other potential biological pathways may involve alterations of adipokines associated with cancer growth such as leptin and adiponectin. Leptin is positively correlated with body mass index [25] and is decreased by CR [10], [18]. Furthermore, both *in vivo* and *in vitro* studies demonstrate the ability of leptin to increase colon cancer cell growth [26]. Adiponectin is increased in CR and decreased in obesity [10], and has been inversely associated with colon cancer risk and progression [27], [28], [29]. Finally, obesity has been linked with increased (and CR with decreased) inflammation [6], [10], although to our knowledge the effects of DIO and CR on colon carcinogenesis, systemic hormones and cytokines, and microRNAs (short RNA molecules that can regulate multiple mRNAs) have not previously been compared.

The current study is (to our knowledge) the first to study the effects of both sides of the energy balance equation (CR and DIO) in a mouse model of chemically-induced colon carcinogenesis. In addition, the potential underlying roles of IGF-1, inflammation, adipokines, and microRNAs in the energy balance-colon carcinogenesis relationship were also tested.

## Materials and Methods

### Animal Study Design

All procedures involving animals were approved and monitored by the National Cancer Institute Animal Care and Use Committee. All diets were purchased from Research Diets, Inc. New Brunswick, NJ) (Table 1). One hundred thirty-two 10-week-old male FVB mice (Charles River, Wilmington, MA) were used in this study. Mice were initially placed on a control diet (modified AIN-76A semipurified diet, catalog #D12450B) and fed *ad libitum*. The colon cancer specific carcinogen, azoxymethane (AOM) (Sigma, St. Louis, MO) was administered via intraperitoneal (i.p.) injections once a week for 5 weeks at a dose of 10 mg/kg beginning at 10 weeks of age. One week after the final AOM injection, mice were individually housed and randomized (n = 44/diet group) for the remainder of the study to: 1) continue the control diet fed *ad libitum*, which provides 3.8 kcal/g; 2) switch to 30% CR diet (catalog #D0302702); or 3) switched to a DIO diet, fed *ad libitum* (catalog #D12451), which provides 5.2 kcal/g with 45% kcal fat. CR mice received a modified formulation of control diet such that daily aliquots of food provided 70% of the mean daily caloric consumption (and 100% of the vitamins, minerals, fatty acids and amino acids) of control mice. Water was provided *ad libitum*.

**Table 1:** Diet composition.

	CR Diet		Control Diet		DIO Diet	
	gm%	kcal%	gm%	kcal%	gm%	kcal%
Protein	27	29	19.2	20	24	20
Carbohydrate	54	57	67.3	70	41	35
Fat	6	14	4.3	10	24	45
**Ingredient**	**gm**	**kcal**	**gm**	**kcal**	**gm**	**kcal**
Casein, 30 Mesh	200	800	200	800	200	800
L-Cystine	3	12	3	12	3	12
Corn Starch	162.9	652	315	1260	72.8	291
Maltodextrin 10	35	140	35	140	100	400
Sucrose	197.9	792	350	1400	172.8	691
Cellulose, BW200	50	0	50	0	50	0
Soybean Oil	25	225	25	225	25	225
Lard	20	180	20	180	177.5	1598
Mineral Mix S10026	10	0	10	0	10	0
DiCalcium Phosphate	13	0	13	0	13	0
Calcium Carbonate	5.5	0	5.5	0	5.5	0
Potassium Citrate, 1 H2O	16.5	0	16.5	0	16.5	0
Vitamin Mix V10001	10	40	10	40	10	40
Choline Bitartrate	2	0	2	0	2	0
FD&C Yellow Dye #5	0.025	0	0.05	0	0	0
FD&C Red Dye #40	0	0	0	0	0.05	0
FD&C Blue Dye #1	0.025	0	0	0	0	0

### Ethics Statement

This study was conducted in accordance with the recommendations in the Guide for the Care and Use of Laboratory Animals of the National Institutes of Health. All procedures were approved by the Institutional Animal Care and Use Committee of the National Cancer Institute (Protocol #: ASP-05-097).

Body weight and food consumption measures were recorded weekly. In addition, fat mass, lean mass, and percent body fat measures were determined in a randomly selected cohort of ten mice per dietary group at the beginning of the study and after ten weeks of dietary treatment via dual-energy X-ray absorptiometer (DEXA) (GE Medical Systems, Madison, WI). Glucose tolerance testing was also conducted after ten weeks of dietary treatment in a randomly selected cohort of ten mice per group. Mice were fasted overnight, baseline blood glucose levels were determined, and glucose was injected i.p. at a dose of 1.5 mg glucose/gram body weight. Blood was then collected via tail nick at 15, 30, 60, 90, and 120 minutes after the glucose injection to measure glucose levels via a glucometer.

Twelve mice per dietary treatment were euthanized with carbon dioxide 5 and 10 weeks after the last AOM injection to monitor colon tumor development and to collect tissue at intermediate timepoints. The remaining 20 animals per dietary treatment were euthanized 20 weeks after the last AOM injection. Calorie-restricted mice were fed daily so there was no significant length of diet restriction at the time of euthanasia. At each of the 3 timepoints, blood was collected and centrifuged following 30 minutes at room temperature, and serum collected and frozen for subsequent analyses. In addition, at 5 and 10 weeks, the colons from 6 mice per dietary group were removed from each mouse, stretched on filter paper and placed in 10% buffered formalin overnight for tumor enumeration. The colons from the remaining 6 mice per group were scraped to obtain colon mucosa, which was snap-frozen in liquid nitrogen. At the final timepoint, 20 weeks after the final AOM injection, colons were removed from each mouse, stretched on filter paper and placed in 10% buffered formalin overnight. After 24 hours, colons were placed in 70% ethanol.

### Tumor visualization

Colons were stained with methylene blue to enumerate the number of tumors. Tumors were counted independently by 2 blinded investigators. After tumors were counted, colons were embedded in paraffin, and sectioned onto coated slides for immunohistochemical analysis.

### Circulating cytokine analysis

Serum samples from all mice were sent to Millipore (Billerica, MA) for analysis using their mouse cytokine multiplex assay which analyzed 10 cytokines, including granulocyte macrophage colony stimulating factor (GM-CSF), interferon-gamma (IFN-g), interleukin 1-beta (IL-1b), interleukin-2 (IL-2), interleukin-4 (IL-4), interleukin-5 (IL-5), interleukin-6 (IL-6), interleukin-10 (IL-10), interleukin-12 (IL-12), and tumor necrosis factor-alpha (TNF-a). Each sample was run in duplicate and the average was used. Cytokine levels below 3.2 pg/ml were considered nondetectable.

### Circulating IGF-1, IGFBP-3, and adipokine analysis

Total serum IGF-1 concentrations were measured in 25 microliter samples using a commercially available enzyme-linked immunosorbent assay (ELISA) kit (R&D Systems, Minneapolis, MN). Insulin, leptin, and resistin were measured using the MILLIPLEX MAP Mouse Serum Adipokine Panel (Millipore) per the manufacturer's instructions. Adiponectin was measured using the mouse single plex adiponectin kit (Millipore) per the manufacturer's instructions. Finally, IGFBP-3 was measured using the Milliplex MAP Mouse IGF Binding Protein Panel for IGFBP-3 (Millipore) per the manufacturer's instructions. Each sample was run in duplicate and the average was used.

### Immunohistochemistry

Immunohistochemistry was performed as previously described [30], [31]. Briefly, slides were deparaffinized, rehydrated, and then microwaved-heated for antigen retrieval in Antigen Retrieval Solution (Vector Laboratories, Inc., Burlingame, CA) for 20 minutes. Endogenous peroxidases were blocked and sections were washed in phosphate-buffered saline containing 0.1% Triton X-100 for 20 minutes to reduce nonspecific binding. Next, sections were blocked with Vectastain Blocking Serum from the Vectastain Elite ABC Kit (Vector Laboratories, Burlingame, CA) for 20 minutes, incubated with the primary antibody (1∶500, proliferating cell nuclear antigen (PCNA), Santa Cruz Biotechnology, Inc.) overnight at 4°C. After several washes, sections were treated with a biotinylated anti-goat IgG for PCNA for 30 minutes at room temperature, treated with an avidin and biotinylated horseradish peroxidase complex, and stained with the chromogen, 3,3′-diaminobenzidine (DAB) (Vector Laboratories, Burlingame, CA) for 1 minute. Sections were counterstained with Vector's Hematoxylin QS Nuclear Counterstain for 45 seconds, dehydrated, and placed in xylene before adding Permount and a coverslip.

### Tdt-mediated dUTP Nick End Labeling (TUNEL) Staining

Sections were used to evaluate apoptosis with the ApopTag Kit (Serologicals Corporation, Norcross, GA) according to the manufacturer's protocol and as previously described [30], [31]. Briefly, sections were deparaffinized, rehydrated, and pretreated with Proteinase K. Next endogenous peroxidases were quenched, sections were equilibrated, and incubated with the terminal deoxynucleotidyl transferase TdT enzyme in order to add digoxigenin-labeled nucleotides to the 3′-OH ends of the DNA. The reaction was stopped and sections were incubated with digoxigenin peroxidase conjugate. The sections were incubated with peroxidase substrate, counterstained with methyl green, washed in 100% butanol, dehydrated, and coverslipped.

### MicroRNA analysis

Total RNA was extracted from frozen scraped colon mucosa according to the manufacturer's instructions for use of the TRIzol reagent (Invitrogen, Carlsbad, CA). RNA integrity was assessed using the Agilent 2100 Bioanalyzer RNA 600 nano assay (Agilent Technologies, Santa Clara, CA). MicroRNA microarray profiling was conducted as previously described [32]. Five mg of total RNA was labeled and hybridized to the microRNA microarray (Ohio State microRNA microarray version 4.0, Columbus, OH). Microarray data was deposited into Gene Expression Omnibus (accession number GSE56025).

Quantitative RT-PCR was used to validate specific microRNAs. The microRNAs that were validated included: hsa-miR-16; mmu-let-7f; mmu-miR-351; has-miR-150; has-miR-425; hsa-miR-196a; hsa-miR-138; and mmu-miR-155 (Applied Biosystems, Foster City, CA). Taqman MicroRNA assays (Applied Biosystems) were used according to the manufacturer's instructions in a 7500 real-time RT-PCR system (Applied Biosystems). Mouse specific small nuclear (sn)/small nucleolar (sno), snoRNA 202, snoRNA 234, and snoRNA 142 endogenous controls were used as the normalization controls (Applied Biosystems). All assays were performed in triplicate.

### Statistical analyses

Percent body fat, tumor multiplicity, cytokine levels, IGF-1, IGFBP-3, insulin, and adipokines were analyzed using an ANOVA for overall differences in body fat among the 3 dietary treatments. To determine which groups were significantly different from one another a Bonferroni post hoc test was conducted. Bodyweight and glucose tolerance testing were analyzed using repeated measures ANOVA. For immunohistochemical analysis, the staining index was calculated based on the number of positive cells divided by a total number of 1000 cells counted. The staining index was averaged for the 3 dietary treatments and compared using a one-way ANOVA. All values were reported as significant if the p-value was less than 0.05. All statistical tests were conducted using Stata Statistical Software Version 11 [33]. For the microarray analysis, the statistical software R 2.5.0 (R Foundation for Statistical Computing, Vienna, Austria) was used to remove probes that had higher background intensities than foreground and to remove probes with inconsistent measurements across the quadruplicates. Data was normalized by locally weighted scatter plot smoothing LOESS and then imported into Biometric Research Branch (BRB) array tools 3.5.0 (http://linus.nci.nih.gov/BRB-ArrayTools.html). Class comparison analysis using paired t tests were used to detect microRNAs that were differentially expressed among the dietary interventions (p<0.001). Real-time PCR data was analyzed using the comparative C_T_ method and fold differences compared to the control group were reported.

## Results

### Body weight and composition

**Figure 1:**
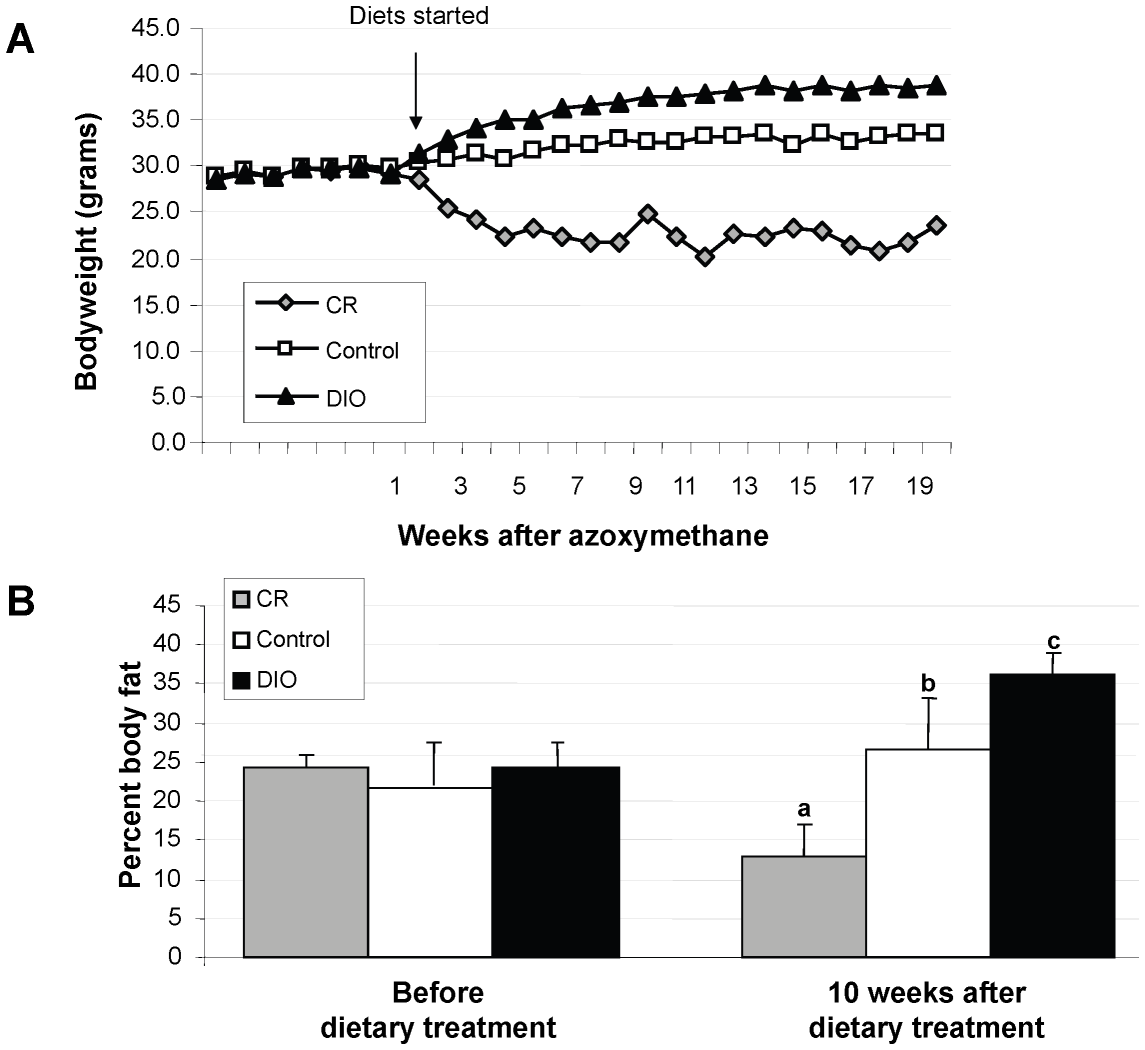
A. Bodyweights in calorie restricted (CR), control, and diet-induced obesity (DIO) FVB mice. Bodyweight was measured weekly in all three dietary interventions. Each data point represents the mean of all the mice in that dietary group. B. Body fat in CR, control, and DIO dietary treatments. Dual-energy X-ray absorptiometer (DEXA) scans were conducted at the beginning of the study and after ten weeks of dietary treatment. Each bar represents the mean of ten mice per group. One-way analysis of variance (ANOVA) at the beginning of the study, p = 0.78. One-way ANOVA at ten weeks, p<0.001. *a*, *b*, and *c* represent dietary treatments that are significantly different from one another (p<0.05) as determined by Bonferroni test.

Ten-week-old male FVB mice were utilized in an AOM model of colon carcinogenesis to determine the effect of CR and DIO on colon tumor development and alterations in underlying biological pathways. Bodyweight was monitored throughout the study (Figure 1A). DIO mice had the highest body weight while CR mice had the lowest body weight. In addition, percent body fat was determined through DEXA scans before the dietary interventions and after the dietary interventions began (Figure 1B). Nine weeks after the start of the dietary intervention, body fat was significantly different among all 3 groups (p<0.0001). Furthermore, the mean percentage of body fat among CR mice was 13.7% lower compared to the control mice (p<0.001) while the mean percentage of body fat among DIO mice was 9.4% higher compared to the control mice (p = 0.002).

**Figure 2:**
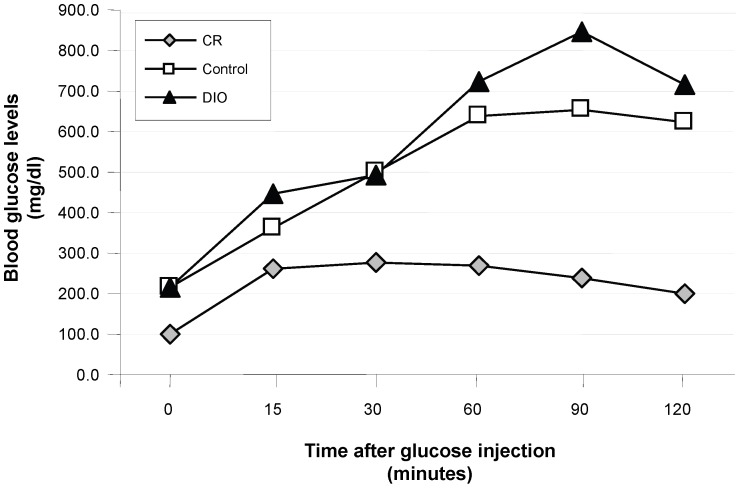
Glucose tolerance testing in CR, control, and DIO mice after ten weeks on dietary treatment. Each data point represents the mean of ten mice per dietary treatment.

Glucose tolerance testing was conducted midway through the study to determine if the model accurately represented obesity in humans (Figure 2). After fasting overnight, blood glucose levels were measured in the mice over a period of 2 hours. DIO mice had higher levels of blood glucose which remained elevated across the 120-minute timecourse compared with the control and (to a greater extent) CR animals. This is consistent with an obesity-associated impairment and a CR-induced improvement, in glucose tolerance.

### Colon Tumor Development

**Figure 3:**
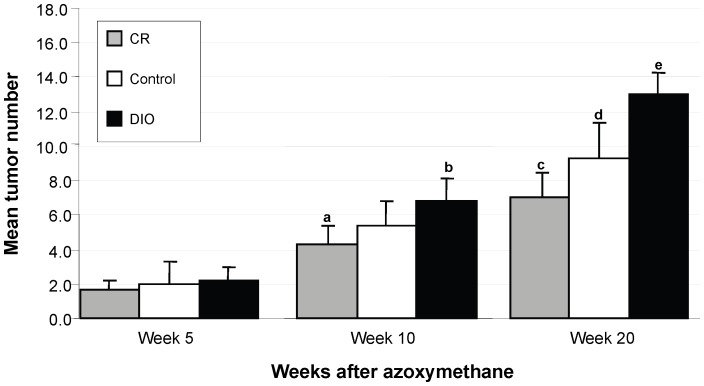
Tumor multiplicity among CR, control, and DIO mice. The total number of tumors was counted along the entire length of the colon after methylene blue staining. Bars represent the average number of tumors per colon in all three dietary groups (n = 12 at 5 weeks, n = 12 at 10 weeks, n = 20 at 20 weeks). Five weeks, one-way ANOVA, p = 0.63. Ten weeks, one-way ANOVA, p = 0.01. Twenty weeks, one-way ANOVA, p = 0.0001. *a* and *b* represent dietary treatments that are significantly different from one another (p = 0.009) as determined by Bonferroni test. *c*, *d*, and *e* represent dietary treatments that are significantly different from one another (p<0.05) as determined by Bonferroni test.

Colon tumor development was assessed at 3 timepoints during the study (Figure 3). The first timepoint was 5 weeks after the last injection of AOM and no difference was observed among the dietary treatments in colon tumor number (p = 0.63). By 10 weeks, however, there was an overall difference in tumor number among the groups (p = 0.01). However, only the CR and DIO mice were significantly different from each other (p = 0.009) with the DIO mice having an average of 2.5 more tumors than the CR mice. At the final timepoint of the study (20 weeks), colon tumor number was significantly different among all 3 groups (p = 0.0001). The DIO mice had a significantly higher number of colon tumors compared to the control (13.0±1.3 compared to 9.2±1.8, p = 0.01). In contrast, the CR mice had significantly fewer colon tumors (6.9±2.3, p<0.001) than controls. Histological examination demonstrated that the majority of tumors were adenocarcinomas among all the dietary treatments. There were no significant differences in the percentage of tumors that were adenocarcinomas between any of the dietary treatments (data not shown).

### Cytokine Expression

**Figure 4:**
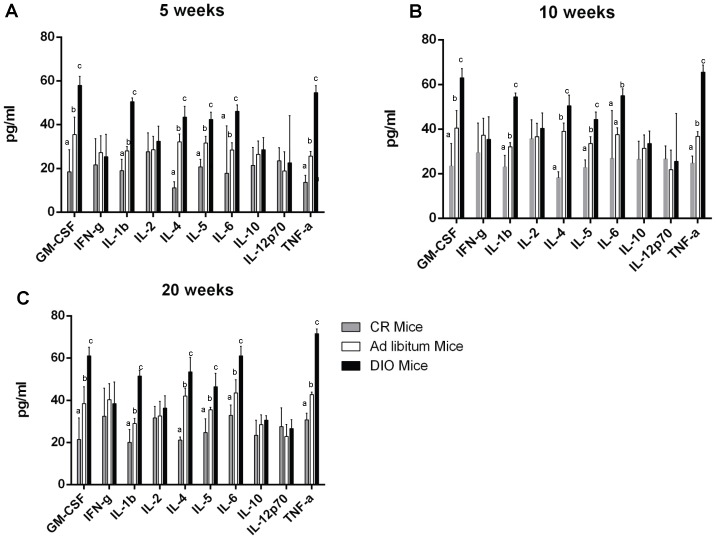
Systemic inflammation in CR, control, and DIO mice. Systemic inflammation was measured by assessing the levels of ten cytokines in the serum of the mice **A**) five, **B**) ten, and **C**) twenty weeks after the last injection of AOM. Bars represent the mean for each cytokine in each dietary group (n = 12 for **A** and **B** and n = 20 for **C**).

Levels of 10 cytokines were examined in the mice in all 3 dietary treatment groups at 5, 10, and 20 weeks after the last injection of AOM (Figure 4). Among the 10 cytokines, 6 were significantly altered at the 5 week timepoint. Levels of TNF-a, IL-1b, IL-4, IL-5, IL-6, and GM-CSF were all significantly higher in the DIO mice compared to the control mice (p<0.001). In addition, levels of all 6 cytokines were significantly lower in the CR mice compared to the control mice (p<0.001). Furthermore, these 6 cytokines remained significantly different at both 10 and 20 weeks after AOM.

### Systemic Hormone Levels

**Figure 5:**
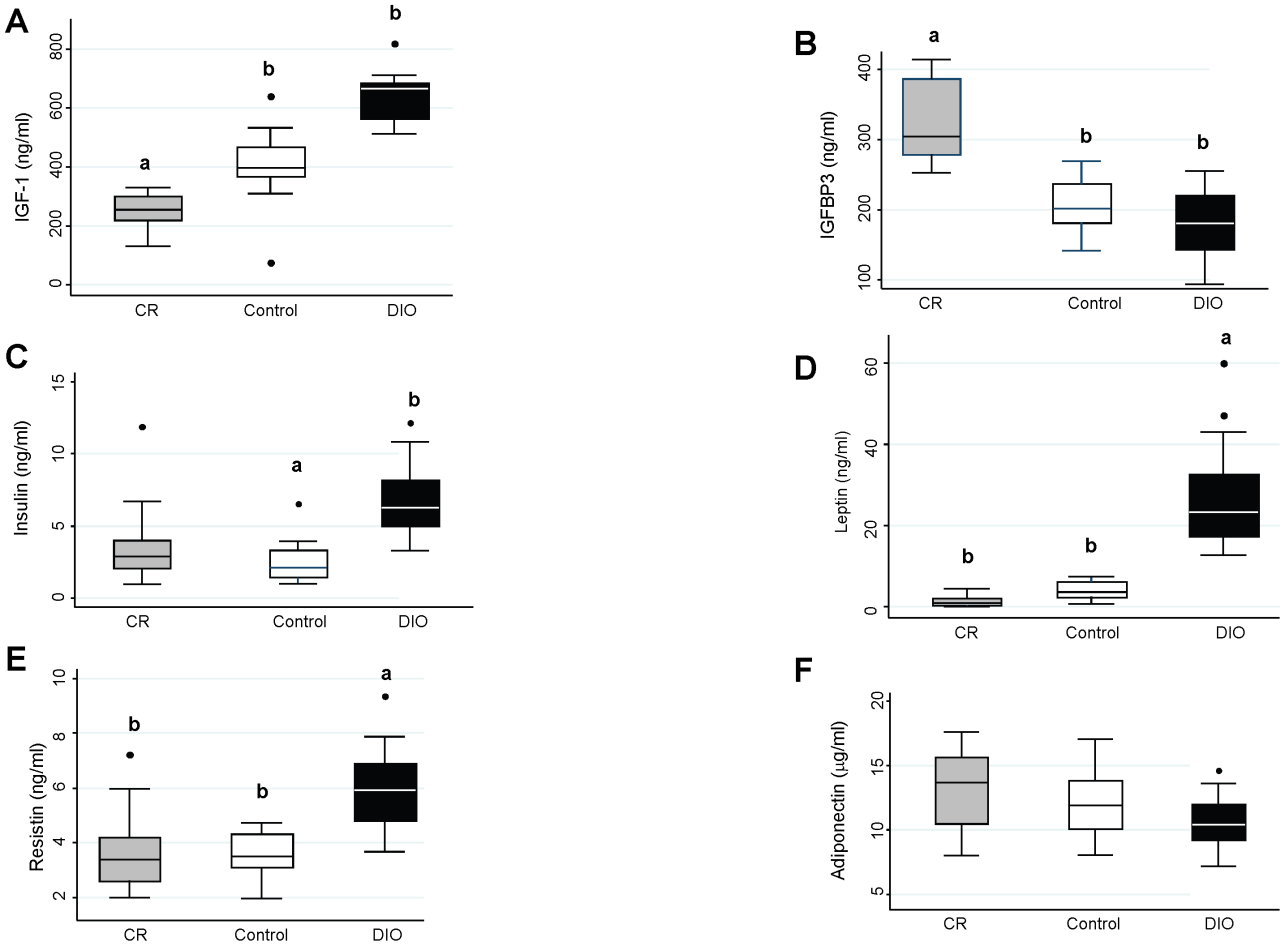
Adipokine, IGF-1, IGFBP-3, and insulin levels among CR, control, and DIO mice. Levels of **A**) IGF-1, **B**) IGFBP-3, **C**) insulin, **D**) leptin, **E**) resistin, and **F**) adiponectin were measured in the serum of the mice ten weeks after the last injection of AOM. Each boxplot represent the mean and range (n = 20). Overall differences among groups were determined using a one-way ANOVA. For **A**) IGF-1, p = 0.002; **B**) IGFBP-3, p<0.001; **C**) insulin, p = 0.002; **D**) leptin, p<0.0001; **E**) resistin, p = 0.001; and **F**) adiponectin, p = 0.14. *a*,*b*, and *c* represent dietary treatments that are significantly different (p<0.05) from one another as determined by a Bonferroni test.

Levels of IGF-1, IGFBP-3, and several adipokines related to obesity and cancer were examined in the serum of the mice 10 weeks after the last AOM injection (Figure 5). Levels of IGF-1 were significantly different among the 3 dietary treatments (p = 0.002) (Figure 5A). DIO mice had the highest level of IGF-1 (665.4±16.2 pg/ml) while CR mice had significantly lower levels of circulating IGF-1 (247.4±15.3 pg/ml) (p<0.001). In addition to levels of IGF-1, levels of IGFBP-3 were lowest among the DIO mice (190.4±50.1 ng/ml) and highest among the CR mice (302.7±46.8 ng/ml) (p<0.001) (Figure 5B). DIO mice also demonstrated significantly higher levels of circulating insulin (6.1±2.8 ng/ml) compared to the control mice (2.4±1.2 ng/ml) (p = 0.002) although there was no statistical significance when compared to the CR mice (3.4±2.2 ng/ml) (p = 0.17) (Figure 5C).

Levels of the adipokine leptin were significantly higher among the DIO mice (22.3±1.5 ng/ml) compared to both the control mice (4.0±2.0 ng/ml) (p<0.001) and the CR mice (1.3±1.2 ng/ml) (p<0.001) (Figure 5D). There was no difference in leptin levels between the CR mice and the control mice (p = 0.81). Similarly, levels of resistin were significantly higher among DIO mice (6.0±1.9 ng/ml) compared to the control mice (3.6±0.8 ng/ml) (p = 0.003) and the CR mice (3.6±1.2 ng/ml) (p = 0.004) (Figure 5E), however there was no significant difference between the CR mice and the control mice (p = 1.0). Finally, there was no difference in adiponectin levels among all 3 dietary treatments (p = 0.14).

#### Proliferation and apoptosis

**Figure 6:**
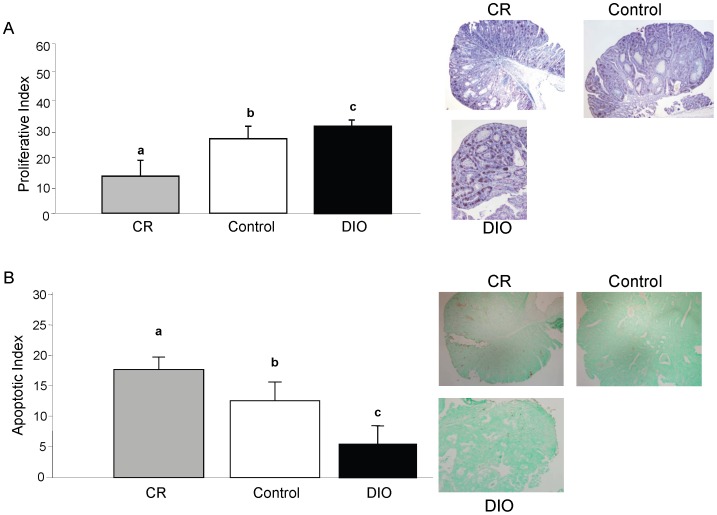
Cellular proliferation and apoptosis in CR, control, and DIO mice 20 weeks after the last AOM injection. **A**) Proliferation was assessed via immunohistochemistry for proliferating cell nuclear antigen (PCNA). A field of 1000 cells was counted for each animal and the proliferative index represents the percentage of cells that stained positive for PCNA. The bars represent the average proliferative index for each dietary group (n = 20). **B**) Apoptosis in the tumors of LID versus wildtype mice 20 weeks after the last AOM injection. Apoptosis was assessed via TUNEL staining. A field of 1000 cells was counted for each animal and the apoptotic index represents the percentage of cells that stained positive and also appeared apoptotic based on cell morphology. The bars represent the average apoptotic index for each dietary group (n = 20). *a*,*b*, and *c* represent dietary treatments that are significantly different (p<0.05) from one another as determined by a Bonferroni test.

Proliferation was investigated 20 weeks after the last AOM injection by examining PCNA levels via immunohistochemistry (Figure 6A). The CR mice had the lowest proliferative index (14.2%±2.5), while the DIO mice had the highest (30.7%±2.1) (p<0.001). In addition, apoptosis was studied in the colon tumors of these mice 20 weeks after the last AOM injection using a TUNEL assay. The apoptotic index was significantly higher in the CR mice (16.8%±1.5) compared to the control mice (12.3%±2.0) and the DIO mice (5.8%±1.9) (p<0.001) (Figure 6B).

### MicroRNA expression

**Figure 7:**
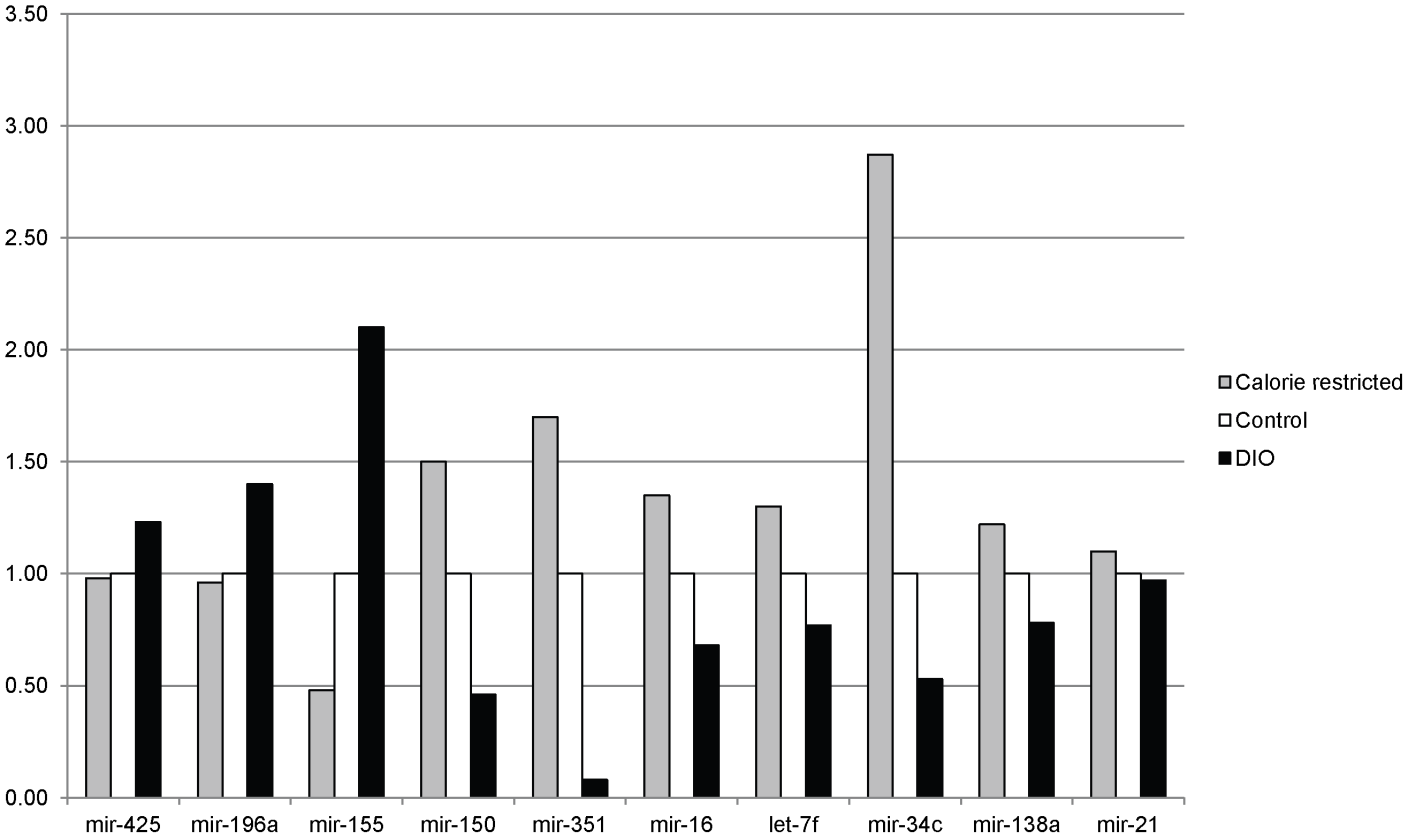
Real time PCR of microRNAs to validate microarray. Ten miRs were validated using real-time PCR. They included miR-425, miR-196a, miR-155, miR-150, miR-351, miR-16, let-7f, miR-34c, miR-138a, and miR-21. Results were analyzed using the comparative C_T_ method and reported as fold differences compared to the control group. Each bar represents the mean of six animals.

A preliminary investigation into possible alterations in microRNA (miR) expression was conducted using RNA samples isolated from colon mucosa 10 weeks after the last injection of AOM (Table 2). Eight miRs were significantly upregulated in the colons of the DIO mice while 10 were significantly downregulated. From these 18 miRs, nine of them were selected (based on previous reports demonstrating their role in carcinogenesis) for validation using quantitative real-time PCR. Of the 9 that were chosen for validation, the fold-changes were confirmed via quantitative real-time PCR (Figure 7). The validated miRs that were significantly upregulated among the DIO mice included miR-425, miR-196, and miR-155. In addition, 6 miRs were validated that were significantly downregulated among the DIO mice. These included miR-150, miR-351, miR-16, let-7, miR-34, and miR138.

**Table 2:** Obesity is associated with differences in microRNA expression.

Probe from Microarray	Mature miR	False Discovery Rate (%)	Fold Change	p-value
**MicroRNAs with increased expression among the DIO mice compared to control**				
**mmu-mir-425-p**	miR-425	2.9	1.59	0.00358
mmu-mir-100-a	miR-100	3.4	1.74	0.00074
mmu-mir-194-1-a	miR-194	3.4	1.65	0.00812
mmu-mir-378-3p/422b-a	miR-378-3p	3.4	1.78	0.00109
mmu-mir-718-p	miR-718	3.4	1.66	0.00147
**mmu-mir-196-a-2-a**	miR-196a	3.4	1.96	0.00155
mmu-mir-669-a	miR-669a	3.4	1.73	0.00157
**mmu-mir-155-a**	miR-155	4.7	2.50	0.00096
**MicroRNAs with reduced expression among the DIO mice compared to control**				
**mmu-mir-150-a**	miR-150	<1.0	0.41	0.00011
**mmu-mir-351-a**	miR-351	2.1	0.37	0.00018
**mmu-mir-16-2-a**	miR-16	3.4	0.64	0.00044
mmu-mir-694-p	miR-694	3.4	0.56	0.00053
**mmu-let-7f-1-a**	let-7	3.4	0.60	0.00118
mmu-mir-682-a	miR-682	3.4	0.62	0.00125
mmu-mir-133a*-5p-a	miR-133a	3.4	0.71	0.00147
**mmu-mir-34c-p**	miR-34c	4.0	0.71	0.00200
**mmu-mir-138-a**	miR-138	4.6	0.26	0.00259
mmu-mir-133a-1-3p-a	miR-133a	4.6	0.63	0.00265

## Discussion

To our knowledge, this is the first study to use both CR and DIO in the AOM mouse model of colon carcinogenesis, along with a preliminary attempt to identify potential mechanisms underlying energy balance modulation of colon tumor development. We observed that a 30% CR diet was effective in producing a lean phenotype while a commonly used high fat DIO regimen was effective in producing an obese phenotype in FVB mice. Many diet-induced obesity models use a 60% kcal high fat diet while the current study used a 45% kcal high fat diet, which in our view is more physiologically relevant to human diets in the US and many other developed countries. There are also numerous mouse studies on obesity and cancer that use genetically modified animals [34], [35], [36], [37]. However, the genetic changes that exist in these animals (usually mutations in leptin or its receptor) that result in hyperphagia and obesity are rare in humans, and the obese phenotype in these mice is severe. Our goal was to model human obesity in our mice as closely as possible.

Although obesity has been associated with increased colon cancer risk, there have been few DIO mouse models established which mimic the human condition and investigate the effect on colon tumorigenesis. Our study demonstrated the ability of a DIO regimen involving a 45 kcal% fat diet to not only induce an obese phenotype, but also increase the number of colon tumors. Furthermore, we also established the effects of energy balance modulation across the spectrum of phenotypes, from lean to control to obese, on colon tumorigenesis. Specifically, a DIO regimen resulted in a significantly higher number of colon tumors and these tumors had increased levels of proliferation and decreased levels of apoptosis compared to both the control and, to a greater extent, the CR mice. Animals were sacrificed at a specific timepoint in the study, therefore, we were unable to assess the effect of energy balance on survival, however, based on our past studies, a reduction in tumor number is consistently associated with extended survival.

Obesity, like cancer, is a condition that likely affects multiple biological pathways simultaneously. Therefore, we initiated investigations into several pathways that have been previously suggested to be associated with obesity and/or colon cancer. Inflammation was one of these pathways. Several researchers have demonstrated that obesity increases inflammation, thereby contributing to colon cancer development [38], [39]. Our study examined the systemic effect of obesity on inflammation by examining levels of several cytokines in the serum. Similar to previous studies, we observed that obesity increased inflammatory cytokine levels and this was correlated with tumor development [36], [38]. Furthermore, this elevation occurred soon after dietary modification and remained elevated, suggesting that this increase was due to the obese phenotype and not just an increase due to increased tumor development. Of these cytokines, proinflammatory cytokines, including TNF-a and IL-6, have been demonstrated to activate the NFkB pathway which is then associated with tumor progression [40]. Therefore, this is another potential pathway involved in the association of obesity and colon cancer and warrants future study. We did not examine inflammation specific to the colon but plan to conduct further studies focused in that area.

Insulin, IGF-1, and IGFBP-3 have also been linked with colon cancer in both in vitro and in vivo studies [19], [30]. Furthermore, these metabolic factors have been suggested to be associated with BMI and obesity [19]. We observed that IGF-1 was increased in the obese animals while IGFBP-3 was decreased, consistent with increased bioavailability of IGF-1 in obese mice. These alterations were at a timepoint when the number of colon tumors was not significantly different between dietary treatment groups. Therefore, it is reasonable to assume these changes were due to obesity and may have contributed to colon tumor growth. Future studies will involve examining the role of obesity in mice that are deficient in IGF-1 to determine the IGF-1-dependence of energy balance effects on colon tumor development. Although we did not examine specific downstream molecules in the insulin/IGF-1 pathway due to tissue limitation, we did examine the end effects of cellular proliferation and apoptosis. The obese animals which had increased levels of IGF-1 had corresponding increases in proliferation in the tumor and decreases in apoptosis suggesting an activation of the IGF-1 pathway. Future studies will further examine the role of the IGF-1 pathway, more specifically, activation of Akt and mTOR pathway components.

Leptin and adiponectin have been associated with colon cancer and are related to BMI [22], [26]. Similar to previous studies we observed increased levels of leptin and non-significantly decreased levels of adiponectin in DIO animals relative to controls [10], [41]. Similar to insulin, IGF-1, and IGFBP-3, these adipokines were measured ten weeks after the last injection of AOM. Therefore, it is reasonable to hypothesize that these changes were due to alterations in body fat and may have also contributed to colon tumor promotion. Not only has leptin been associated with obesity, but it has also been demonstrated to increase levels of cytokines such as IL-6 and IL-1b, cytokines that were also elevated in our study [37]. Other studies have also demonstrated the ability of leptin to enhance expression of proinflammatory cytokines [42], [43], therefore our study supports these findings. Adiponectin has been observed to have a protective role in colon cancer, perhaps through its anti-inflammatory properties [44], [45]. We did not observe a significant decrease in adiponectin in our study, although levels did decrease in the DIO animals. Further studies in our animal model will more fully investigate the role of leptin and adiponectin in regulating cytokine expression in the colon.

Finally, our study examined differences in microRNA expression between all 3 groups. To our knowledge, this is the first study examining energy balance interventions and microRNA expression in a murine colon cancer model. This was a preliminary investigation into microRNA expression in this model. Our intent was to gain some preliminary data on differential microRNA expression that we could then use for follow-up studies that were hypothesis-driven. Loss of miR-34c has been observed in many types of cancer along with loss of let-7F and miR-16[46], [47]. Increased levels of microRNA-150 have been demonstrated to be associated with increased apoptosis and decreased proliferation [48], corresponding with what we observed in our study. In contrast, microRNA-155 and 196 were upregulated in our study among the mice on a DIO regimen. Previous studies demonstrate that miR-155 overexpression is present in lung, breast and colon tumors [49], [50]. In addition, miR-155 has been associated with intestinal inflammation and ulcerative colitis [51]. Furthermore, increased miR-196 has been associated with increased colon metastases and phosphorylation of Akt [52]. While we did not examine levels of Akt directly, Akt is downstream of IGF-1 which was increased in our study. In addition, an established downstream effect of Akt activation is proliferation and cell survival. We did observe that animals on the DIO diet had increases in proliferation and decreases in apoptosis consistent with alterations in the IGF-1/Akt/mTOR pathway [53], [54].

In summary, the current study is (to our knowledge) the first to: 1) use a physiologically relevant 45% kcal fat diet to induce obesity and to directly compare the effects of DIO and CR (which induce an obese and lean phenotype, respectively) on colon carcinogenesis; 2) assess energy balance effects on a broad panel of systemic hormones, growth factors, and cytokines; and 3) evaluate the relationships between energy balance modulation, miR expression and colon carcinogenesis. We successfully demonstrated that the DIO regimen resulted in an increase in colon tumor development, while CR decreased colon tumor development. We further established that these diets altered several biological pathways previously hypothesized to be involved in obesity and cancer, including inflammation, IGF-1, adipokines, and microRNAs. We conclude that components of the IGF-1 signaling and inflammatory cytokine pathways (and potentially the cross-talk between these pathways) should be considered in future studies of targets and strategies for preventing obesity-related colon cancer.
